# Amplicons, Metagenomes, and Metatranscriptomes from Sediment and Water

**DOI:** 10.1128/mra.01230-22

**Published:** 2023-03-01

**Authors:** Madison R. Newman, Darlenys Sanchez, Anna M. Acosta, Bernadette J. Connors

**Affiliations:** a Science Department, Dominican University New York, Orangeburg, New York, USA; University of Southern California

## Abstract

High fecal indicator bacterium (FIB) counts in water have been found to correlate with high sediment FIB counts. To determine the other bacterial populations in common between the two substrates, sediment and water samples from suburban waters known to be impacted by stormwater runoff were examined using next-generation sequencing.

## ANNOUNCEMENT

Sites in the lower Hudson River watershed were initially chosen based on data obtained from Hudson Riverkeeper (1), as well as one site that was not included in their analyses (Spring Valley, NY). Riverkeeper is a nonprofit environmental organization dedicated to the protection of the Hudson River and its tributaries. Several sites that were sampled that had high failure rates, as determined by whether the samples collected previously by Riverkeeper met the EPA guideline for safe swimming. Water samples (1 L) were collected in sterile Nalgene bottles that were first rinsed with creek water three times prior to being fully submerged. The water was filtered through sterile nitrocellulose filters (pore size, 0.22 μm). Nearshore creek bed sediment (5 mL) was collected by submerging closed, sterile 15-mL conical tubes and then releasing the seal to collect the sediment, making all efforts to minimize water flow into the collection bottle.

DNA and RNA were extracted from 0.25 g of each sample using the ZymoBIOMICS DNA/RNA miniprep kit. Metagenomic libraries were constructed using the Nextera XT DNA library prep kit (Illumina). Metatranscriptomic libraries were prepared with 100 ng of total RNA using the NEBNext Ultra RNA kit for double-stranded cDNA synthesis and metatranscriptome library preparation. Libraries between 250 and 400 bp were purified on a 2% agarose gel using a Qiagen QIAquick gel extraction kit. Sequencing was performed on an Illumina NextSeq 550 instrument at Wright Labs (Huntingdon, PA, USA) to produce 2 × 150-bp reads. FastQC v0.11.9 (2) and fastp v0.22.0 (3) were used to check and filter the raw data. The microbial and functional features of the samples were determined by annotating the paired sequence data using HUMAnN v2 (4), with sequences identified as belonging to Homo sapiens removed using KneadData v2 (5). The UNIREF90 (UniProt/UniRef database v2014_07) genes from the functional annotation were mapped to KEGG v56 orthologs (6). Identification of bacteria to the species level was conducted by collating the HUMAnN v2 taxonomic identifications. Default parameters were used for all software unless otherwise specified.

For 16S rRNA gene microbial community profiling, PCR was performed on DNA extracts based on the Earth Microbiome Project’s 16S rRNA gene amplification protocol (7). The PCR products were pooled and purified after separation on a 2% agarose gel. The pooled libraries were quality checked using a 2100 Bioanalyzer high-sensitivity DNA analysis kit (Agilent Technologies). Sequencing was conducted by Wright Labs using Illumina MiSeq v2 chemistry with paired-end 250-bp reads. Demultiplexing was performed using BCL2fastq v2.19.0.316 (Illumina) with default settings. The demultiplexed paired-end reads were processed using QIIME2 v2021.2 (8) with the DADA2 plug-in (9). The preformatted Silva SSU nonredundant (NR) 99 full-length rRNA gene sequence reference database was used to assign taxonomy (10, 11).

Table 1 details properties of the three ’omics data sets, including the relative abundance of select bacterial taxa. The taxa presented are those that had a relative abundance of >1% and were differentially represented in the two substrates. Although not shown in Table 1, several members of *Bacteroides* were identified in Moturis and Spring Valley water. *Prevotella*, *Parabacteroides*, *Ruminococcus* (*Blautia*), *Bifidobacterium*, and *Faecalibacterium*, which are all feces-associated bacteria (12–15), were only identified in Moturis water samples analyzed by shotgun metagenomics. Together, these genera represent 6.89% of the identified bacteria (classified and unclassified) and 23.5% of the classified bacteria. Based on the differential relative abundance of taxa in soil and water from the six sites, these data may be used to inform future efforts toward microbial source tracking.

**TABLE 1:** Properties of the ’omics data sets

Site	GPS coordinates	Substrate	NGS^*a*^ type	No. of reads	SRA accession no.	Predominant taxa (% relative abundance)^*b*^
Sparkill	41.025363, −73.927466	Sedminet	16S	13,381	SRR22221596	*Comamonadaceae* (3.4), *Dechloromonas* (3.4)
			MG	223,012	SRR22221592	Unclassified (100)
			MT	6,832,234	SRR22221579	Unclassified (100)
Sparkill	41.025363, −73.927466	Water	16S	100,660	SRR22221595	*Comamonadaceae* (39.2), *Polynucleobacter* (8.9), *Dechloromonas* (1.1)
			MG	7,240,270	SRR22221591	*Polynucleobacter* (7.14), unclassified (91.8)
			MT	11,090,123	SRR22221578	*Polynucleobacter* (4.23), unclassified (95.8)
Blauvelt Arm	41.056438, −73.944968	Sedminet	16S	73,024	SRR22221584	*Comamonadaceae* (5.9), *Dechloromonas* (2.8)
			MG	928,109	SRR22221590	Unclassified (100)
			MT	12,648,884	SRR22221577	Unclassified (100)
Blauvelt Arm	41.056438, −73.944968	Water	16S	64,698	SRR22221573	*Comamonadaceae* (6.8), *Polynucleobacter* (0.2), *Dechloromonas* (1.5)
			MG	2,549,195	SRR22221589	Enterobacter (9.5), unclassified (90.5)
			MT	11,661,858	SRR22221576	Unclassified (100)
Marsh	41.038606, −73.915210	Sedminet	16S	61,323	SRR22221566	*Comamonadaceae* (0.9)
			MG	3,507,848	SRR22221588	*Sulfuricella* (15.5), unclassified (84.5)
			MT	17,329,967	SRR22221575	Unclassified (100)
Marsh	41.038606, −73.915210	Water	16S	76,475	SRR22221565	*Comamonadaceae* (5.5), *Polynucleobacter* (0.1)
			MG	4,019,881	SRR22221587	*Flavobacteria* (4), *Halothiobacillus* (2), unclassified (92.7)
			MT	24,687,649	SRR22221574	Unclassified (100)
Moturis	41.015904, −73.937346	Sedminet	16S	80,759	SRR22221562	*Comamonadaceae* (6.5), *Dechloromonas* (1.8)
			MG	3,582,410	SRR22221583	*Thiobacillus* (2.1), unclassified (97.9)
			MT	10,130,592	SRR22221570	Unclassified (100)
Moturis	41.015904, −73.937346	Water	16S	72,182	SRR22221561	*Comamonadaceae* (11.3), *Polynucleobacter* (3.5), *Dechloromonas* (0.5)
			MG	5,739,788	SRR22221582	Enterobacter (4.3), *Eubacterium* (3.7), Acinetobacter (2.2), Klebsiella (5.3), *Polynucleobacter* (5.8), *Ruminococcus* (4.2), unclassified (70.6)
			MT	14,379,033	SRR22221569	*Polynucleobacter* (4.9), unclassified (95.1)
Spring Valley	41.115367, −74.042263	Sedminet	16S	64,866	SRR22221564	*Comamonadaceae* (5.2), *Dechloromonas* (4.3)
			MG	842,136	SRR22221586	Unclassified (100)
			MT	9,706,868	SRR22221572	Unclassified (100)
Spring Valley	41.115367, −74.042263	Water	16S	75,542	SRR22221563	*Comamonadaceae* (16.7), *Polynucleobacter* (8.1), *Dechloromonas* (1.1)
			MG	3,185,050	SRR22221585	*Polynucleobacter* (8.9), *Megamonas* (2.7), *Microcystis* (1.04), unclassified (95.4)
			MT	14,873,372	SRR22221571	*Polynucleobacter* (4.4), unclassified (86.6)
Rockleigh	41.007620, −73.940000	Sedminet	16S	45,611	SRR22221594	*Comamonadaceae* (6.1), *Dechloromonas* (4)
			MG	13,797,720	SRR22221581	*Rhodopseudomonas* (4.2), *Sulfuricella* (2.2), *Thiobacillus* (2.2), unclassified (91.4)
			MT	9,838,983	SRR22221568	*Thiobacillus* (9.9), unclassified (90.1)
Rockleigh	41.007620, −73.940000	Water	16S	80,467	SRR22221593	*Comamonadaceae* (35.5), *Polynucleobacter* (4.3), *Dechloromonas* (1.4)
			MG	4,825,003	SRR22221580	*Polynucleobacter* (7.3), unclassified (92.7)
			MT	10,021,470	SRR22221567	*Polynucleobacter* (4.4), unclassified (95.5)

a NGS, next-generation sequencing; MG, metagenomic; MT, metatranscriptomic.

b Only select bacterial taxa are reported in this table.

### Data availability.

The raw sequencing data are available at the NCBI Sequence Read Archive (SRA) under BioProject accession number PRJNA898587. The SRA accession numbers are listed in Table 1.
